# Clinical Notes from Private Practice

**Published:** 1849-11

**Authors:** George L. Upshur

**Affiliations:** Norfolk, Va., Member of the American Medical Association


### MEDICAL EXAMINER,

AND

### RECORD OF MEDICAL SCIENCE.

NEW SERIES.—NO. LIX.—NOVEMBER, 1849.

---

### ORIGINAL COMMUNICATIONS.

---

*Clinical Notes from Private Practice.* By GEORGE L. UPSHUR, M. D., Norfolk, Va., Member of the American Medical Association.

(Concluded from October number.)

*Fever after Child-birth.*—Case 1. Mrs. S., before mentioned; did perfectly well until May 3d. During the night before, she had some soreness of the limbs, and thirst, which prevented her from sleeping as well as usual. She attributed this to a sudden change in the weather, by which she became chilled. At my morning visit, I found her lying with the feet drawn up, languid, thirsty and restless, with general aching of the limbs, and considerable tenderness of the abdomen upon pressure, and upon any motion of the lower extremities. The pulse was rather feebler than the day before, but not more frequent than natural. The bowels well moved twice on the 2d, by two Pil. Cathart. Comp. Prescribed hot fomentations to the abdomen, to be followed by sinapisms and hops wrung out of hot water. She took internally a tablespoonful of this mixture:

℞—Sp. Æther. Sulph. c. f.℥i.
Tr. Opii Camph.
Tr. Lav. c. *aa* f.℥ii.
Aquæ Camph. f.℥iiss. M.

I was summoned to see her again at 10 o'clock, two hours after my first visit. Found her just getting over a severe rigor, with increased thirst, headache; pulse small, compressible, 120; and intense pain over the whole abdomen, which was greatly increased by the slightest pressure, or by the least motion of the lower extremities, *especially rotation of the thighs.* Prescription—continue hops and mustard. A tablespoonful every two hours of the following:

| | | |
|---|---|---|
| ℞—Potass. Nitrat. | ℨi. | |
| Morph. Sulph. | gr. ss. | |
| Aquæ | f.℥iv. | M. |

*Afternoon.*—Patient feels more comfortable; less pain upon pressure; profuse perspiration; pulse 104, soft; no headache; anorexia; restless; continue treatment.

*May 4th. Morning.*—Entire absence of fever; much less pain; can turn from side to side without much inconvenience; appetite. At the afternoon visit I found my patient rapidly convalescing.

Case 2.—Mrs. B., a delicate lady, æt. 25, was delivered of her third child on the 1st of May, after a labor of fifteen hours. The expulsion of the placenta was followed by considerable hemorrhage, sufficient to pale the cheek and induce a feeling of faintness. The next day, 2d, she imprudently walked across the room to a sofa, in order to have her bed arranged; and returned to bed without being greatly fatigued. May 4th, after being awake all the previous night with the child, she was seized with chill, followed by high fever; pulse 130, small and feeble; and intense pain radiating from the hypogastrium over the whole abdomen, which was greatly increased by pressure, motion, deep inspiration or cough. There was also *suppression of the lochia,* with thirst, nausea, hot, but moist skin, and decubitus upon the back, with the thighs well flexed upon the abdomen. Prescribed nitrate of potash and morphia, and mustard and hops, as in Mrs. S.'s case. In the afternoon the pain was less, but no abatement in the febrile action.

*May 5th.* Less fever and less pain; did not sleep well last night. Prescription: ℞. Quiniæ Sulph. gr. xvi; Pulv. Opii gr. v; Pulv. Ipecac. gr. iv. M.; Pil. viii. S. one pill every two hours; ice in moderate quantities; continue the topical applications. At the afternoon visit, the patient had taken four pills, and was decidedly better. Continue treatment.

*May 6th.* Found Mrs. B. free from fever, and nearly free from soreness. Rested well last night; lochia abundant. Discontinued medicine; she recovered rapidly.

*Case* 3.—On the 2d of May, Mary, colored, æt. 22, the mother of three children, miscarried at the sixth month. She lost a good deal of blood two days before the expulsion of the fœtus, but not enough seriously to impair her strength. On the 4th, two days after the abortion, she was seized in a manner similar to Mrs. S., though less violently, and was treated in the same way. At the expiration of 36 hours she was free from fever and pain, and has not had an unpleasant symptom since.

*Remarks.*—The three cases just detailed, presented all of the prominent symptoms of puerperal peritonitis; rigors, followed by high fever; intense pain in the abdomen; inability to rotate the lower extremities; dorsal decubitus, with the thighs well flexed; thirst and rapid pulse. Gordon, Lee, Hey, Armstrong, and our own Dr. Meigs, who have written the best articles upon this important disease, all mention these symptoms as pathognomonic of the affection. Meigs is so explicit upon the subject as to say—"For many years past, I have, perhaps, never called upon one of my accouchées, within four or five days after delivery, without asking her to show me whether she could draw up her knees without pain. If she can do so, I am always content"—and by implication, if she cannot, he is not content. In all of my cases, the patient could not rest with the feet extended, and the slightest contraction of the psoas and iliacus muscles was productive of great pain. But after all, were these cases of puerperal peritonitis? I think not. They recovered too rapidly under a treatment comparatively mild. Either the active antiphlogistic treatment recommended by Meigs and others, is unnecessary in puerperal fever, or these were not cases of that disease. But if the symptoms in every case were such as are pronounced in puerperal fever, how is it possible to make a correct diagnosis in such cases? Had I studied the cases reported by Gordon, Hey, &c., as carefully before I saw the cases herein reported, as I have since studied them, I should have pursued a different treatment, and, I doubt not, in case 2, at least, with a different result.

There are no morbid conditions so similar in their symptoms, and yet so widely different in their real nature, as *irritation* and *inflammation*. The first accomplishes no good purpose in the economy, but wastes its energy and impairs its power of resistance; the last has for its object the reparation of an injury, or the elimination of some noxious agent, which would destroy the harmonious play of the human machine. In the language of Boerhaave, it is the "*vis vitæ conans evertere mortem*;" and though often unsuccessful, and sometimes, in its zeal, the destroyer of that which it would preserve, its *aim* is invariably to do good. It is true, that irritation is sometimes of so high a grade as to bring it nearly within the bounds of true inflammatory action; while on the other hand, there are forms of inflammation which exhibit that want of balance between the great nervous centres, which renders it near akin to irritation; but generally the line of demarkation is sufficiently distinct.

It is extremely important to make a correct diagnosis; for irritation does not bear the evacuant treatment, while inflammation calls loudly for it. If a physician is called to a case of severe headache, accompanied by double vision, tinnitus aurium, flushed face, &c., and without further inquiry, proceeds to bleed the patient freely, he would be astonished to find syncope supervene after the loss of a few ounces, and all the symptoms increased in severity; but if he should be informed that, a few hours before, the patient had lost a large amount of blood from the uterine vessels, he would at once see that he had to deal with what are termed *hemorrhagic* symptoms, and would pursue an opposite treatment, no matter how pointedly these symptoms seemed to indicate an *inflammatory* condition of the brain.

The symptoms in my cases I attributed to irritation, the result of hemorrhage, fatigue and loss of sleep, and therefore adopted the soothing, tonic treatment, instead of the antiphlogistic. I believe if Mrs. B., case 2, had been bled and purged freely, she would have died.

*Ptyalism accompanied by Acute Dysentery.*—On the 30th of May, Mr. M., a confirmed dyspeptic, applied to me for relief from a serious diarrhœa, from which he had been suffering for a day or two. I gave him the following prescription: ℞. Calomel gr. vi., Capsici gr. xii., Camph gr. x., Opii gr. iii., Ol. Menthæ gtt. vi. M. Pil. 6. S. One pill every two hours. He took four of the pills, and being relieved, discontinued them. On the 1st of June he was profusely salivated, but walking about, and free from fever. Was called to see him on the 3d. Found him feverish, nauseated, and with loose bowels, the dejections being liquid and accompanied by considerable pain. ℞. Tr. Camph., Tr. Opii *aa* ℨij., Ol Menthæ gtt. x. M. S. Forty drops every hour until relieved. He sent for me in the night; was suffering from tenesmus; pulse full, hard, and bounding; frequent vomiting; pain upon pressure along the course of the colon: bowels moved every ten minutes, and the discharges very bloody. Took *twenty-nine* ounces of blood from the arm, by measurement; ordered 12 Spanish leeches to the abdomen. ℞. Acid. Nitrosi ʒi., Tr. Opii. gtt. xl., Aquæ Camph. f.℥vii. M. S. One-fourth of this mixture every four hours. Visited him in eight hours; leeches took ten ounces of blood; all the symptoms were improved; nausea gone; discharges scarcely tinged with blood; pulse soft and compressible; pain nearly disappeared. The patient recovered rapidly.

I have several times this year seen dysentery while the system was under the mercurial influence. In such cases Hope's nitrous acid mixture will be found invaluable; and, indeed, I believe it will be acknowledged to be the best remedy in any case of acute dysentery. For the past seven years, I have prescribed it in this disease to the exclusion of almost every other remedy, and have scarcely ever been disappointed in it. The failures with it, are due, I believe, to its being given in too small doses.

